# Survival analysis of women breast cancer patients in Northwest Amhara, Ethiopia

**DOI:** 10.3389/fonc.2022.1041245

**Published:** 2022-12-19

**Authors:** Bereket Feleke, Lijalem Melie Tesfaw, Aweke A. Mitku

**Affiliations:** ^1^ Department of Statistics, Bahir Dar University, Bahir Dar, Ethiopia; ^2^ Schools of Mathematics, Statistics and Computer Science, College of Agriculture Engineering and Science, University of KwaZulu-Natal, Durban, South Africa

**Keywords:** accelerated failure time, breast cancer, frailty, survival analysis, women, associated factors

## Abstract

**Introduction:**

Breast cancer, the most common cause of cancer death and the most frequently diagnosed cancer among women worldwide, ranks as the second cause of death next to lung cancer. Thus, the main objective was to assess the factors that affect the survival time of breast cancer patients using the shared frailty model.

**Methods:**

A retrospective study design was used to collect relevant data on the survival time of breast cancer patients from the medical charts of 322 breast cancer patients under follow-up at the Felege Hiwot Comprehensive Specialized Hospital (FHCSH). The data were explored using the Cox proportional hazard model, the accelerated failure time model, and shared frailty models. The model comparison was done using AIC and BIC. As a result, the Weibull gamma shared frailty model had a minimum AIC and BIC value.

**Result:**

From a total of 322 patients, about 95 (29.5%) died and 227 (70.5%) were censored. The overall mean and median estimated survival times of breast cancer patients under study were 43.7 and 45 months, respectively. The unobserved heterogeneity in the population of clusters (residence) as estimated by the Weibull-gamma shared frailty model was 0.002 (p-value = 0.000), indicating the presence of residential variation in the survival time of breast cancer patients. The estimated hazard rate of patients who had not had recurrent breast cancer was 0.724 (95% CI: 0.571, 0.917) times the estimated hazard rate of patients who had had recurrent breast cancer.

**Conclusion:**

The prevalence of breast cancer was considerably high. Under this investigation, older patients, patients in stages III and IV, anemic and diabetes patients, patients who took only chemotherapy treatment, metastasized patients, patients with an AB blood type, patients with a positive breast cancer family history, and patients whose cancer was recurrent had high death rates. Patient characteristics such as age, stage, complications, treatment, metastasis, blood type, family history, and recurrence were significant factors associated with the survival time of women with breast cancer.

## Introduction

Cancer is a disease caused by the uncontrolled division of abnormal cells in a part of the body. It begins when cells start to grow uncontrollably due to genetic changes that impair their normal evolution. It can develop almost anywhere in the body (1). Among numerous types of cancer, breast cancer is the second most common type after lung cancer, each contributing 12.3% of the total number of new cases diagnosed by 18 million people (2, 3), but in 2020 breast cancer became the first and the leading common type of cancer, contributing (11.72%) for breast cancer and (11.43%) for lung cancer and it will be the most deadly disease for females in developed and developing countries if treatment is not initiated at an early stage (4, 5).

Breast cancer is the most diagnosed cancer among all women worldwide overall and in 140 of the world’s 184 countries, representing one-quarter of all cancers diagnosed in women. It is also the leading cause of cancer deaths among women globally. Although once primarily considered a disease of Western women, 52% of new breast cancer cases and 62% of deaths now occur in economically developing countries (6). It is the first leading cause of cancer death among women in 2020 in Africa, with over 1.1 million cases and 711,429 deaths (Breast Cancer Statistics and Resources, 2020). The most common female malignancy in most African countries is cancer, with the first or second highest incidence and/or mortality rate (7).

The number of deaths from breast cancer in Africa is estimated to double by the year 2050. In sub-Saharan Africa (SSA), breast cancer survival rates are poor, and diagnosis of cancer at earlier stages could prevent deaths (8). African women from sub-Saharan Africa will be found to have a low incidence of breast cancer, yet a higher mortality rate compared to women from developed nations (9). The estimated age-standardized rates for breast cancer incidence in sub-Saharan Africa (which range from 15 to 53 per 100,000 women) are lower than in Western countries (which range from 53 to 102 per 100,000) (10).

In Ethiopian women, breast cancer is the most prevalent type of cancer. Despite the high incidence, Ethiopia’s mortality rate and breast cancer patient survival rates were poorly understood (11). While there is probably very substantive underreporting as rural women seek help from traditional healer seeking, the incidence of breast cancer rate in Ethiopia is low, many are never diagnosed and not reported particularly from the rural side of the country, and there is not much awareness from health care professionals (12). Many patients in Ethiopia are unaware of the signs and symptoms of breast cancer, which leads to delays in reaching an effective diagnosis and care and is often complicated by unclear and inefficient navigation of the health system and, consequently, often presents too late for effective treatment (13, 14). According to various research, the trend of breast cancer over the past 16 years has increased year over year. This increase may be related to a lack of awareness, the hospital’s remote location, and the hospital’s limited capacity for breast cancer diagnostics (15).

Rates of survival with breast cancer have significantly increased all over the world in the past decades. Researchers led by (16), a senior epidemiologist for the American Cancer Society, examine international trends in incidence and death rates of 39 and 57 from all over the world, respectively. In contrast to incidence trends, breast cancer death rates have decreased in most countries (17). Most of the decreasing trends, as well as the most rapid declines, occurred in high-income countries because of improved breast cancer treatment and early detection through mammography. But while death rates are dropping in most countries, the study finds that they have increased in economically transitioning countries (18). In most high-income nations, mortality rates are falling while incidence rates are rising or remaining stable. The actual cause for concern, however, is the rising incidence and fatality rates in developing nations. Increased risk factors brought on by economic growth and urbanization, such as obesity, adoption of a Western-style diet, physical inactivity, delayed childbearing, and shorter nursing length, are most likely to blame for the rise in breast cancer incidence in emerging nations (17, 18).

Research from Vietnam has found that educational status affects the risk of mortality. This is because with a higher level of education there is approximately a 10% risk reduction in death. Marital status has also been found to be a prognostic factor for the mortality of women with breast cancer (17). Age has a significant effect on women’s breast cancer patients. It indicates that the mortality rate for breast cancer is high as age increases (18). A study conducted in the United States on the effects of some risk and prognostic factors contributing to the survival of breast cancer patients showed that tumor size, lymph node metastasis status, and tumor extension had a significant effect on breast cancer survival. Alcohol consumption increases the risk of mortality due to breast cancer. The risk of breast cancer in women is about 7% to 12% for each 10 g (roughly one drink) of alcohol consumed per day (19). A study conducted by Addis Ababa University on the effects of some risk factors contributing to the survival of breast cancer patients shows that stage and pathology type had a significant effect on breast cancer survival (20). Likewise (21), suggests modest positive associations between urbanization and the socioeconomic environment of residential areas and breast cancer incidence.

Several studies have been done on the survival analysis of breast cancer (20, 22, 23). However, the random effects in the model that account for unobserved variability do not include this research. A statistical modeling idea called the frailty method seeks to account for heterogeneity brought on by unmeasured factors. A frailty model is a random effect model for time-to-event data in statistics (24).

When survival data are derived from various groups or when individuals have repeated measures, heterogeneity across individuals should be considered. Numerous issues could arise if heterogeneity is ignored, including an overestimation of the relative hazard rate, inaccurate estimates of the regression coefficients, and estimations of the regression parameters that tend to zero (18, 25). Although semi-parametric and parametric survival models have been the subject of extensive research, accounting for frailty in the model has garnered significant attention more recently. An attempt was made to use frailty models in this investigation. Therefore, the purpose of this study was to use frailty survival analysis to determine the prevalence and associated factors among breast cancer patients.

## Methods

### Study area and study design

The data for this study were collected from the Felege Hiwot Comprehensive Spatialized Hospital (FHCSH), Bahir Dar, Ethiopia. A retrospective cohort study design was carried out by retrieving relevant information from the medical records of BC patients to meet the specified objectives (26).

### Data source

The survival data were extracted from the patient’s chart from the data record office at the Felege Hiwot Comprehensive Specialized Hospital (FHCSH) from January 2018 to January 2022. The study subjects were BC patients diagnosed in different parts of Ethiopia (urban and rural).

The shared frailty models were applied based on their residence. The data chart contains the patient’s history from the entry date up to the discharge date. In this study, breast cancer patients represent the number of patients who follow the clinical treatment up to the discharge date and or who either leave the hospital by any means, or transfer from the hospital to another hospital or die before completing the treatment by any accident other than breast cancer or those who are on treatment. The data were collected by a statistician and public health officer and coded, cleaned, and analyzed using SPSS 20, STATA 16, and SAS 9.4 statistical software.

### Operational definition

#### Stage

Describes the extent or spread of cancer at the time of diagnosis. Cancer’s stage is based on the size or extent of the primary tumor and whether it has spread to nearby lymph nodes or other areas of the body (27, 28). This was done based on authoritative guidelines or references published by the American Joint Committee on Cancer (AJCC) (8, 9).

#### Malignant

Malignant cells grow in an uncontrolled way and can invade nearby tissues and spread to other parts of the body through the blood and lymph system (27, 28).

### Sample size determination

The sampling method is a process by which a representative sample is chosen from a population to use as a sample. There are several plans from which the sample can be picked. With knowledge of the degree of accuracy required, approximate estimates of the sample size can be made for each plan that is considered. For each program, the relative costs and time involved are also compared before making a decision (29).

Many survival studies are designed to compare two alternative therapies, but there may also be information available on the values of such explanatory variables. It was demonstrated by Schoenfeld. Thus, the sample size determination considered in this study was based on (30).

Is given by: $d=4\frac{{\left(z_{\beta }\;+\;z_{\alpha /2}\right)}^{2}}{\theta_{R}^{2}}$ And $n=\frac{d}{p(death)}$ Where n is the sample size required, α is the level of significance or the size of the critical region in hypothesis testing, β is the power of the test, *d*; is the total number of deaths, and θ_R_ is the log hazard ratio. In this study, the power of 80% and the 5% level of significance will be used, and then *Z*_β_ = 0.84 and *Z*_α/2_ = 1.96 from the standard normal distribution table.

The probability of an event occurring and the log-hazard ratio were extracted from (31). This study compared patients at stage I with and without radiotherapy. According to the study, the probability of death was 0.411, and the hazard ratio at stage I with radiotherapy was 3.15 *θ_R_* = log (3.15) = 0.498. Hence, the total sample size is 307 and by considering a 95% confidence interval and a 5% margin of error. Finally, the total sample size is calculated, and we get the final sample size of n = 322.

### Inclusion and exclusion criteria

All people who registered with full information, including study variables of interest on the registration card, were considered eligible for the study. The study included patients who started breast cancer treatment in January 2018 and had at least one follow-up until January 2022. Patients who have incomplete information regarding study variables on their registration cards are not eligible for the study; also, breast cancer patients who dropped out of the study without starting any treatment and the breast cancer patients who died due to accidents are not included in the study.

### Variables considered in the research

#### Outcome variables

The main outcome variable for this study was time to death for breast cancer patients.

#### Independent variables

Several predictors will be considered in this study. They are mentioned here as:

➢ Socio-demographic variables:
  ∘ Age
  ∘ Residence
➢ Clinical factors:
  ∘ Clinical stages
  ∘ Complication
  ∘ Treatment used
  ∘ Pathology type
  ∘ Metastasis
  ∘ Blood type
  ∘ BMI
  ∘ Family history
  ∘ Recurrent status

### Methods of data analysis

#### Survival analysis

Survival analysis is a collection of statistical procedures for data analysis for which the outcome variable of interest is time in years, months, weeks, or days from the beginning of follow-up of an individual until an event occurs. In survival analysis, we usually refer to the time variable as survival time because it gives the time that an individual has “survived” over some follow-up period (32).

#### Non-parametric survival analysis

Non-parametric survival analysis is more widely used in situations where there is doubt about the exact form of distribution. In non-parametric survival analysis, the data are conveniently summarized through estimates of the survival function and the hazard function. The estimation of the survival distribution provides estimates of descriptive statistics. These methods are said to be nonparametric since they require no assumptions about the distribution of survival time. The Kaplan–Meier, Nelson–Aalen, and Life tables are most widely used to estimate the survival and hazard functions (32).

#### Kaplan–Meier estimate of the survival function

The Kaplan–Meier (KM) estimator is the standard non-parametric estimator of the survival function used for estimating survival probabilities from observed survival times, both censored and uncensored (33).

#### Comparison of survivorship functions

When comparing groups of subjects, it is always a good idea to begin with a graphical display of the data in each group.

#### Log-rank test

The log-rank test, sometimes called the Cox–Mantel test, is the most used nonparametric test for comparing two or more groups of survival functions and is the most well-known test statistic.

Therefore, the log-rank test statistic becomes


(1) $$\;Q_{LR}=\frac{{\left[\sum_{i=1}^{m}\left(d_{1i}-\hat{e_{1i}}\right)\right]}^{2}}{\sum_{i=1}^{m}\hat{v_{1i}}}$$

### Semi-parametric survival analysis

#### Cox proportional hazard model

The Cox Proportional Hazard Model is a multiple regression method used to evaluate the effect of multiple covariates on survival (34). A semi-parametric model is proposed for the hazard function that allows the addition of covariates while keeping the baseline hazards unspecified and taking only positive values. This model gives an expression for the hazard at time t for an individual with a given specification of a set of explanatory variables denoted by X, and it is generally given by:


(2) $$h(t,X,\beta )=h_{0}(t)\text{exp}(X\beta )$$

Where *h*_0_ (*t*) is the baseline hazard function at time t, X is the vector of values of the explanatory variables, and *β* = (*β*1, *β*2, … *βk*) is the vector of unknown regression parameters that are assumed to be the same for all individuals in the study, which measures the influence of the covariate on the survival experience.

Note *h*_0_ (*t*) is the hazard function, where all values of the covariates are zero, i.e., *γ*(*X*=0,*β*)=1 .

The ratio of the hazard functions for two subjects with covariate values denoted *X*_1_ *and X*_2_ is given by


(3) $$HR(t_{1},x_{1},x_{2})=\frac{h(t,x_{2},\beta )}{h(t,x_{1},\beta )}=\frac{h_{0}(t)r(x_{2},\beta )}{h_{0}(t)r(x_{1},\beta )}=\frac{r(x_{2},\beta )}{r(x_{2},\beta )}=e^{\beta (x_{2}-x_{1})}$$

Therefore, the hazard ratio (HR) depends only on the function *r*(*X, β*).This model is referred to as the Cox model, the Cox proportional hazards model, or simply the proportional hazards model. The term proportional hazards refer to the fact that the hazard functions are multiplicatively related.

Assumptions:

The baseline hazard depends on t, but not on the covariates *x*_1_, *x*_2_ …, *x*_p._

The hazard ratio, i.e., exp (*β’X*) depends on the covariates *X =* (*x*_1_, *x*_2_ …, _p_) but not on time t.

#### Accelerated failure time model

Although parametric models are very applicable to analyzing survival data, there are relatively few probability distributions for survival time that can be used with these models. The accelerated failure time model is an alternative to Cox PH and parametric models for the analysis of survival time data when the assumption of Cox PH is violated. Unlike the proportional hazards model, the regression parameter estimates from AFT models are robust to omitted covariates. They are also less affected by the choice of probability distribution (35). Under AFT models we measure the direct effect of the explanatory variables on survival time instead of a hazard. This characteristic allows for an easier interpretation of the results because the parameters measure the effect of the correspondent covariate on the mean survival time. The common distributions of the AFT model include exponential AFT, Weibull AFT, log-logistic, log-normal, and gamma distributions.

### Distributions used in AFT models

To be used in an AFT model, a distribution must have a parameterization that includes a scale parameter. The logarithm of the scale parameter is then modeled as a linear function of the covariates.

#### Weibull accelerated failure time model

The Weibull distribution can be parameterized as an AFT model, and they are the only family of distributions to have this property. The Weibull distribution is a very flexible model for time-to-event data. It has a hazard rate that is monotonously increasing, decreasing, or constant (36).

#### Log-logistic accelerated failure time model

One limitation of the Weibull hazard function is that it is a monotonic function of time. However, the hazard function can change direction in some situations. The log-logistic distribution provides the most used AFT model for such situations. The log-logistic distribution is a non-monotonic hazard function that increases at early times and decreases at later times. It is similar in shape to the log-normal distribution, but its cumulative distribution function has a simple closed form, which becomes important computationally when fitting data with censoring (37).

#### Log-normal accelerated failure time

The log-normal distribution is also defined for random variables that take positive values, so it may be used as a model for survival data. If the survival times are assumed to have a log-normal distribution, the baseline survival function and hazard function are given by:

##### Modeling frailty

Using parametric or semi-parametric regression models is an important way to handle heterogeneity. Regression models take a lifetime as the dependent variable and explanatory variables are regressed. Sometimes these models may not provide an adequate fit to the data. One of the reasons is due to the omission of important covariates. Several methods have been developed to model the frailty in survival data in recent years. The generalization of the Cox proportional hazards model (34) is the best and most widely applied model that allows for the random effect by multiplicatively adjusting the baseline hazard function.

Frailty models extend the Cox proportional hazards model by introducing unobserved frailties to the model. In this case, the hazard rate will not be just a function of covariates but also a function of frailties. A frailty model is a random effects model that has a multiplicative effect on the hazard rates of all members of the subgroup. In univariate survival models, it can be used to model heterogeneity among individuals, which is the influence of unmeasured risk factors in a proportional hazards model. In multivariate survival models, the shared frailty model is used to model the dependence between individuals in the group. In multivariate cases, unobserved frailty is common among a group of individuals.

##### Shared frailty models

This frailty model allows individuals in the same group to share the same frailty (38). Due to the assumption that the frailties in each cluster are random, it is also referred to as a mixture model. Given the frailty, it is assumed that each event time in a cluster is independent. The shared frailty model was introduced by (39) without using the notion of frailty and was extensively studied by (40) (41), and (42).

Suppose there are n clusters, and that the *i*th cluster has n_i_ individuals and is associated with an unobserved frailty Z_i_, 1≤ i≤ n. A vector X_ij_ 1≤ i≤ n, 1≤ j ≤ n_i_ is associated with the *j*th complete survival time T_ij_ of the *j*th individual in the *i*th cluster. Conditional on frailties Z_i_, the survival times are assumed to be independent and their hazard functions to be of the form


(4) $$h(t)=h0(t)Z_{i}\exp(\beta 'x_{ij}),i=1,\ldots ,n,j=1,\ldots ,ni$$

where *h*_0_(t) is the baseline hazard function and β is a vector of fixed effect parameters to be estimated. Frailties *Z_i_* are assumed to be identically and independently distributed random variables with a common density function (*z*, θ), where θ is a parameter of the frailty distribution.

#### Frailty distributions

##### Gamma distribution

The Gamma frailty model belongs to the power variance function family (40) and can be expressed in terms of its Laplace transform, from which properties such as mean and variance are easily derived (42) From a computational and analytical point of view, it fits very well with failure data. It is widely used due to its mathematical tractability (43).

Note that if θ >0, there is heterogeneity. So, the large values of θ reflect a greater degree of heterogeneity among groups and a stronger association within groups. The conditional survival function of the gamma frailty distribution is given by (43).


(5) $$S_{Ѳ}(t)={\left[\left(1-Ѳ\ln\left\{S(t)\right\}\right)\right]}^{-1/\theta }$$

The conditional hazard function of the gamma frailty distribution is given by (43):


(6) $$h(t)=h(t){\left[1-\theta \ln\left\{S(t)\right\}\right]}^{-1}$$

(7) $$h(t)=h(t){\left[1-\theta \ln\left\{S(t)\right\}\right]}^{-1}$$

where S(t) and h(t) are the survival and hazard functions of the baseline distributions, respectively.

The variance θ of the frailty term represents heterogeneity among clusters, while the mean is constrained to 1 to make the average hazard identifiable. A larger variance indicates a stronger association within groups.

For the Gamma distribution, Kendall’s Tau (40) measures the association between any two event times from the same cluster in the multivariate case and is given by:


(8) $$\tau =\frac{\theta }{\left(\text{θ}+2\right)},where\;\tau \in (0,1)$$

##### Inverse Gaussian frailty distribution

The inverse Gaussian (inverse normal) distribution was introduced as a frailty distribution alternative to the Gamma distribution by (44) and has been used by (36). Like the gamma frailty model, simple closed-form expressions exist for the unconditional survival and hazard functions; this makes the model attractive. The probability density function of an inverse Gaussian shared distributed random variable with parameter θ >0 is given by


(9) $$f_{z}(zi)=\frac{1}{\sqrt{2\pi }}zi^{-\frac{3}{2}}\text{exp}\left(\frac{-{\left(zi-1\right)}^{2}}{2\theta zi}\right),z>0$$

## Results

### Descriptive statistics

Among a total of 322 breast cancer patients, about 95 (29.5%) experienced the event. When we consider the baseline characteristics of patients, 196 (60.9%) are urban, and the rest are rural (see **Table 1**).

**Table 1:** Descriptive summary of covariate variables for breast cancer patients.

Variable	Categories	Total (%)	Status	
			Censored (%)	Event (%)
**Residence**	Rural	126 (39.1%)	65 (28.6%)	61 (64.2%)
	Urban	196 (60.9%)	162 (71.4%)	34 (35.8%)
**Stage**	1	58 (18.1%)	57 (25.1%)	1 (1.1%)
	2	88 (27.3%)	78 (34.4%)	10 (10.5%)
	3	83 (27.3%)	48 (21.1%)	35 (36.8%)
	4	93 (28.9%)	44 (19.4%)	49 (51.6%)
**Complication**	No	173 (53.4%)	163 (71.8%)	9 (9.5%)
	Yes	150 (46.6%)	64 (28.2%)	86 (90.5%)
**Treatment**	Chemotherapy	154 (47.8%)	129 (56.8.0%)	25 (26.3%)
	Chemotherapy and Surgery	100 (31.1%)	50 (22.0%)	50 (52.6.1%)
	Chemotherapy and Hormonal therapy	68 (21.1%)	48 (21.1%)	20 (21.1%)
**Pathology**	Ductal carcinoma	206 (64.0%)	174 (76.7%)	32 (33.7%)
	Lobular carcinoma	116 (36.0%)	53 (23.3%)	63 (66.3%)
**Metastasis**	No	153 (47.5%)	153 (58.6%)	20 (21.1%)
	Yes	169 (52.5%)	94 (41.4%)	75 (78.9%)
**Blood type**	A	74 (23.0%)	63 (27.8%)	11 (11.6%)
	B	104 (32.3%)	67 (29.5%)	37 (38.9%)
	O	55 (17.1%)	36 (15.9%)	19 (20.0%)
	AB	89 (27.6%)	61 (26.9%)	28 (29.5%)
**BMI**	Obese	18 (5.6%)	13 (5.7%)	5 (5.3%)
	Overweight	68 (21.1%)	43 (18.9%)	26.3 (26.3%)
	Normal	177 (55.0%)	127 (55.9%)	50 (52.6%)
	Underweight	59 (18.3%)	44 (19.4%)	15 (15.8%)
**Family history**	No	203 (63.0%)	174 (76.7%)	29 (30.5%
	Yes	119 (37.0%)	53 (23.3%)	66 (69.5%)
**Recurrent**	No	173 (53.7%)	142 (62.6%)	31 (32.6%)
	Yes	149 (46.3%)	85 (37.4%)	64 (67.4%)

Regarding the baseline stage, 58 (18.1%), 88 (27.3%), 83 (27.3%), and 93 (28.9%) of the patients were in stages I, II, III, and IV, respectively. Among the patients in stages I, II, III, and IV at baseline, 1 (1.1%), 10 (10.5%), 35 (36.8%), and 49 (51.6%) died during the study period, respectively. This shows that most patients who started cancer treatment in stages III and IV had a shorter survival time. Out of the total sample of 322, 112 (32%) patients had associated diseases like anemia, diabetes, hypertension, and other kinds of diseases. Among these patients, 86 (90.5%) experienced the event. Also, **Table 2** shows that, of the total participants, patients who took only chemotherapy treatment had the highest number: 154 (47.8%), followed by patients who took chemotherapy and surgery: 100 (31.1%), and patients who took chemotherapy and hormonal therapy: 68 (21.1%). Among these, 25 (26.3%), 50 (52.6.1%), and 20 (21.1%) experienced the event, respectively (see **Table 1**).

**Table 2:** Multivariable cox proportional hazards regression model analysis.

Variable	Categories	*ф*	St. Error	z	P>z	95%CI	Cof.
**Age**	–	1.028	.012	2.20	0.028	(1.003, 1.053)	.027
**Stage**	1 (ref) 2 3 4	3.973 14.997 11.936	1.065 1.026 1.030	1.30 2.64 2.41	0.195 0.008 0.016	(0.493, 32.055) (2.009, 111.934) (1.586, 89.835)	1.380 2.708 2.480
**Complication**	No (ref) Yes	4.569	.396	3.84	0.000	(2.104, 9.921)	1.519
**Treatment**	Chemotherapy (ref) Chemo&Surgery Chemo&Hormonal	.889 .637	.278 .334	-0.43 -1.35	0.670 0.177	(0.516, 1.531) (0.331, 1.227)	-.118 -.451
**Pathology**	Ductal carcinoma (ref) Lobular carcinoma	1.381	.242	1.34	0.182	(0.860, 2.219)	.323
**Metastasis**	No (ref) Yes	1.709	.282	1.90	0.058	(0.982, 2.973)	.536
**Blood type**	A (ref) B AB O	1.158 2.345 1.158	.368 .416 .375	0.91 2.05 0.39	0.363 0.040 0.695	(0.680, 2.872) (1.038, 5.295) (0.556, 2.415)	.334 .852 .146
**Family history**	No (ref) Yes	1.860	.270	2.30	0.021	(1.096, 3.158)	.621
**Recurrent**	No (ref) Yes	1.926	.237	2.77	0.006	(1.210, 3.064)	.655

**Table 3** revealed the average age of the year’s breast cancer patients included in the study to be 43.8 with a standard deviation of 10.131. This is a minimum age of 26 years, and a maximum age of 75 years.

**Table 3:** Summary of continuous variables for breast cancer patients.

Variable	Maximum	Minimum	Mean	Std. deviation
**Age**	75	26	43.8	10.131

### Non-parametric survival analysis

#### Kaplan–Meier survival estimate for survival time of breast cancer patients

**Figure 1:**
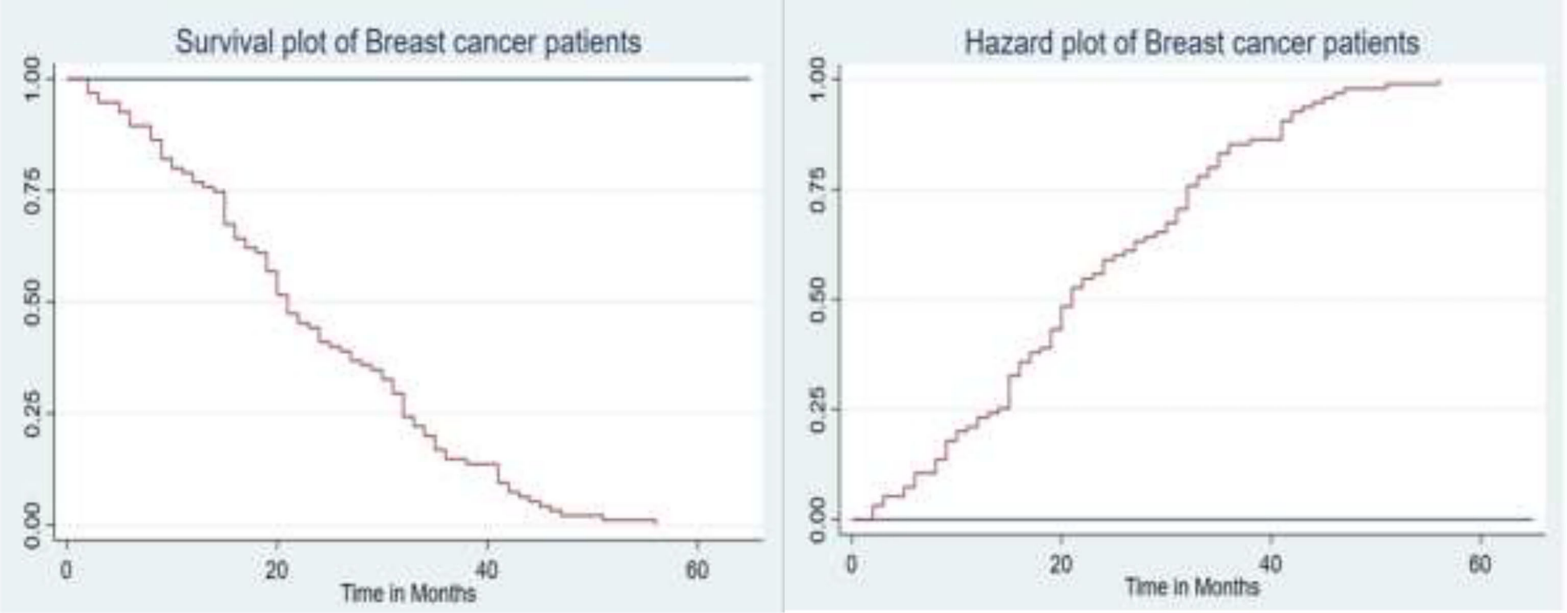
The plot of the overall estimate of Kaplan–Meier survivor function (left) and hazard function (right) of BC patients.

The graph in **Figure 1** (left) shows that most of the deaths occurred in the earlier months of breast cancer treatment and declined in the later months of follow-up. The hazard functions are depicted in **Figure 1** (right), showing that an increase in the hazard rate has a direct relationship to the increase in time.

### Survival function of different categorical groups of covariates

Descriptive graphs of the survivor function would be used for comparing the events experienced at a time by two or more groups and the survival quantities of covariates to describe the survival experience of an individual at specific times. The Kaplan–Meier estimator of the survival curve gives the estimate of survivor function among different groups of covariates to make comparisons. In general, the pattern of one survivorship function lying above another means the group defined by the upper curve has better survival than the group defined by the lower curve.

**Figure 2:**
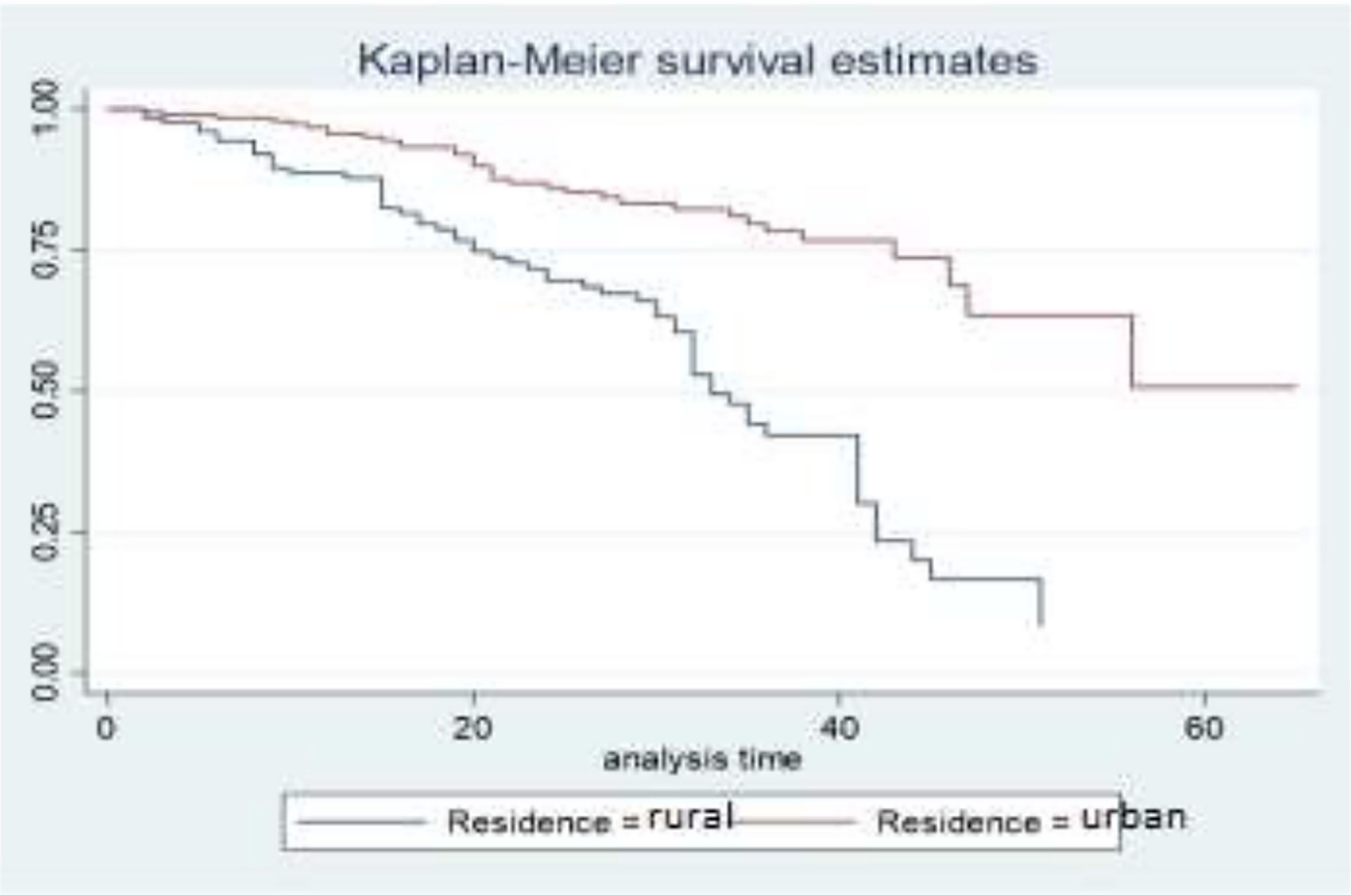
Survival curves by residence.

The gap between the two curves distinguishes the survival distribution of survival time of breast cancer patients by their residence. The differences that are displayed in the survival curve above show that breast cancer patients who come from urban areas have higher survival times when compared to those who came from rural areas. That means BC patients who come from urban areas have higher survival experiences than those who came from rural areas (**Figure 2**).

**Figure 3:**
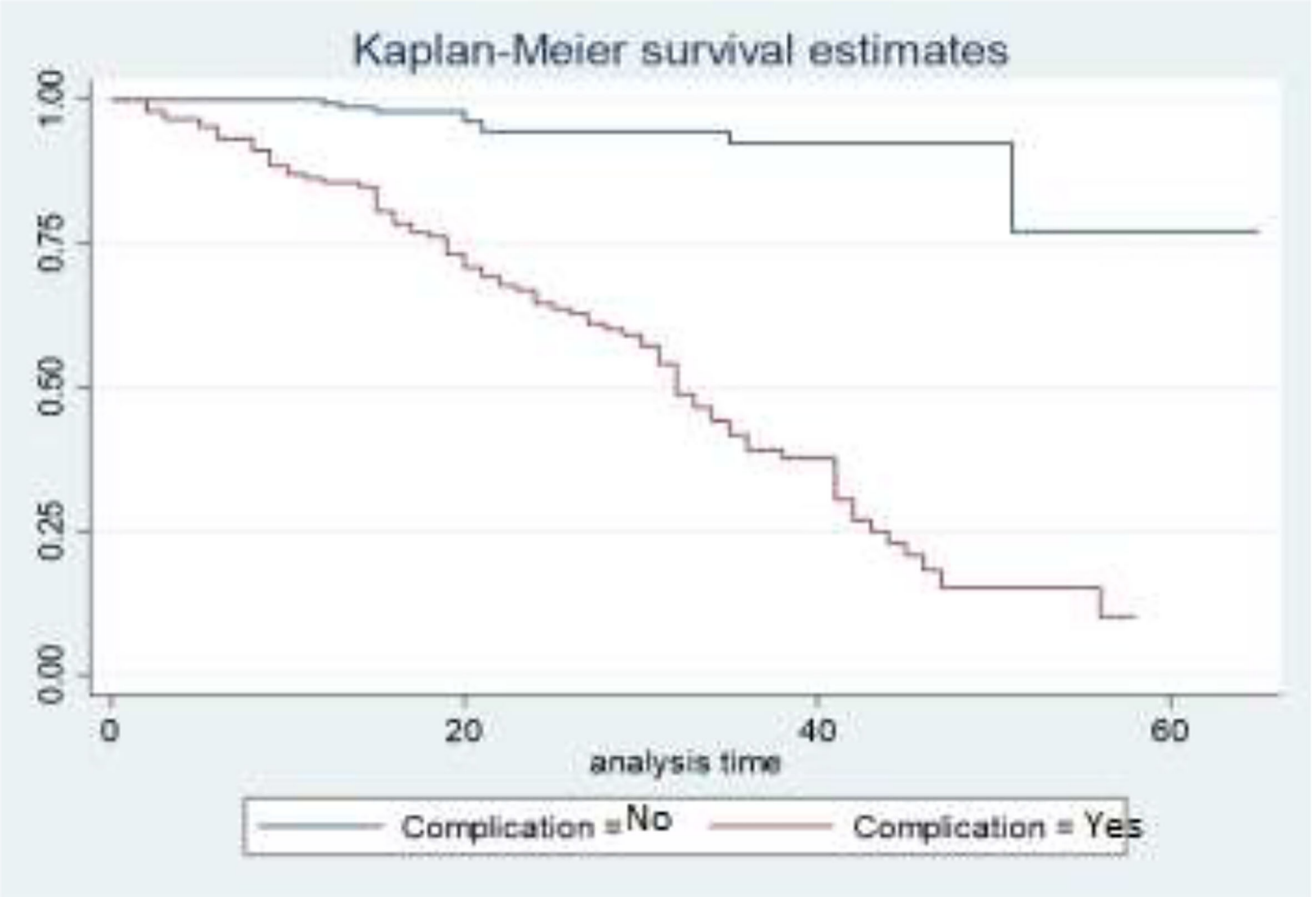
Survival curves of complication status.

The survival curves in **Figure 3** show that the survival experience for BC patients who have complications is lower than the survival experience for BC patients who do not have complications. This means that patients who have associated diseases like anemia, diabetes, and hypertension have higher death experiences compared to patients who have no associated diseases.

**Figure 4:**
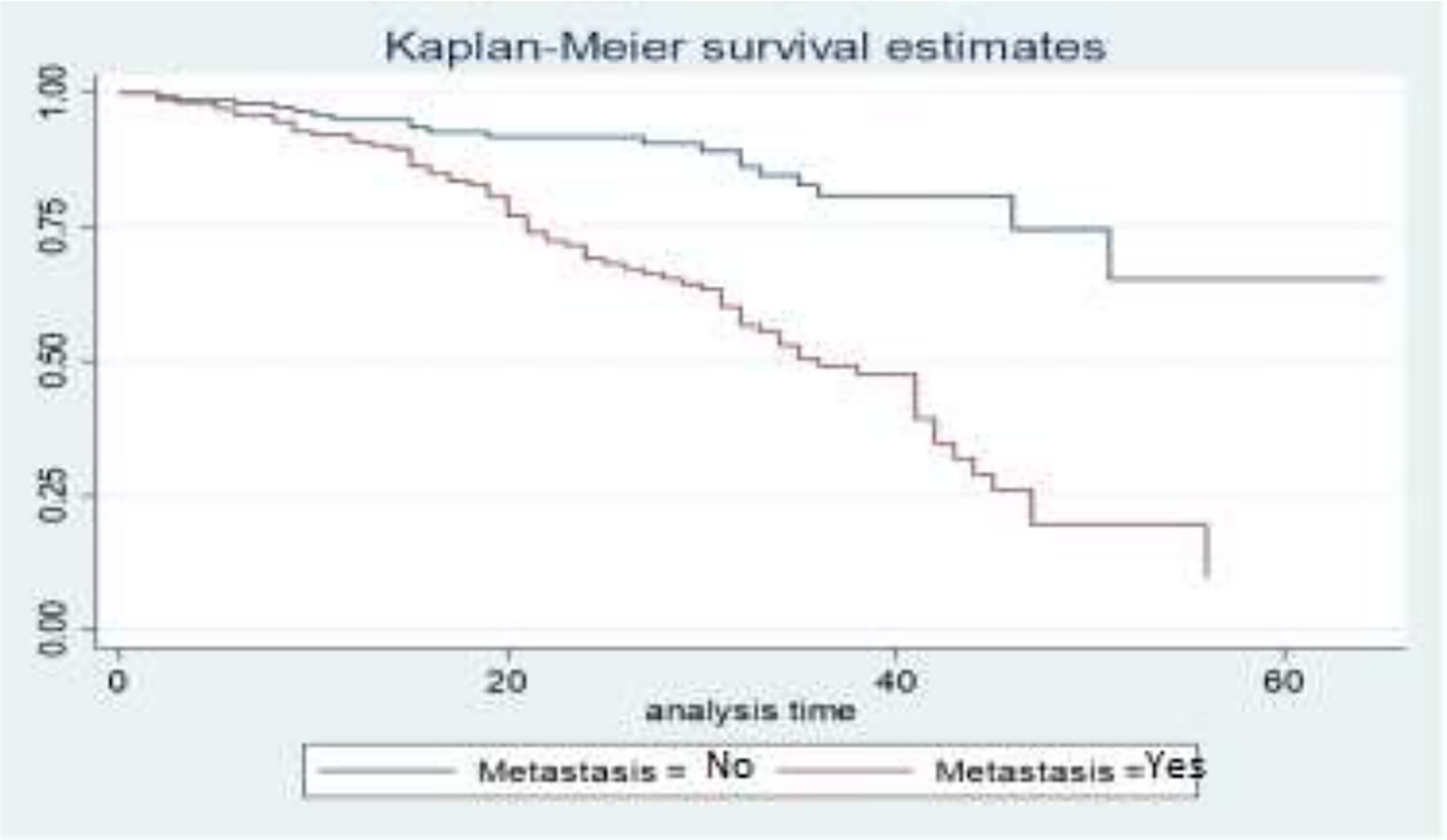
Survival curves of metastasis status.

The survival curves in **Figure 4** show that BC patients whose cancer is not metastasized have a better survival experience than BC patients whose cancer is metastasized. This means that patients who have metastatic cancer have a lower survival rate than patients whose cancer has not metastasized.

**Figure 5:**
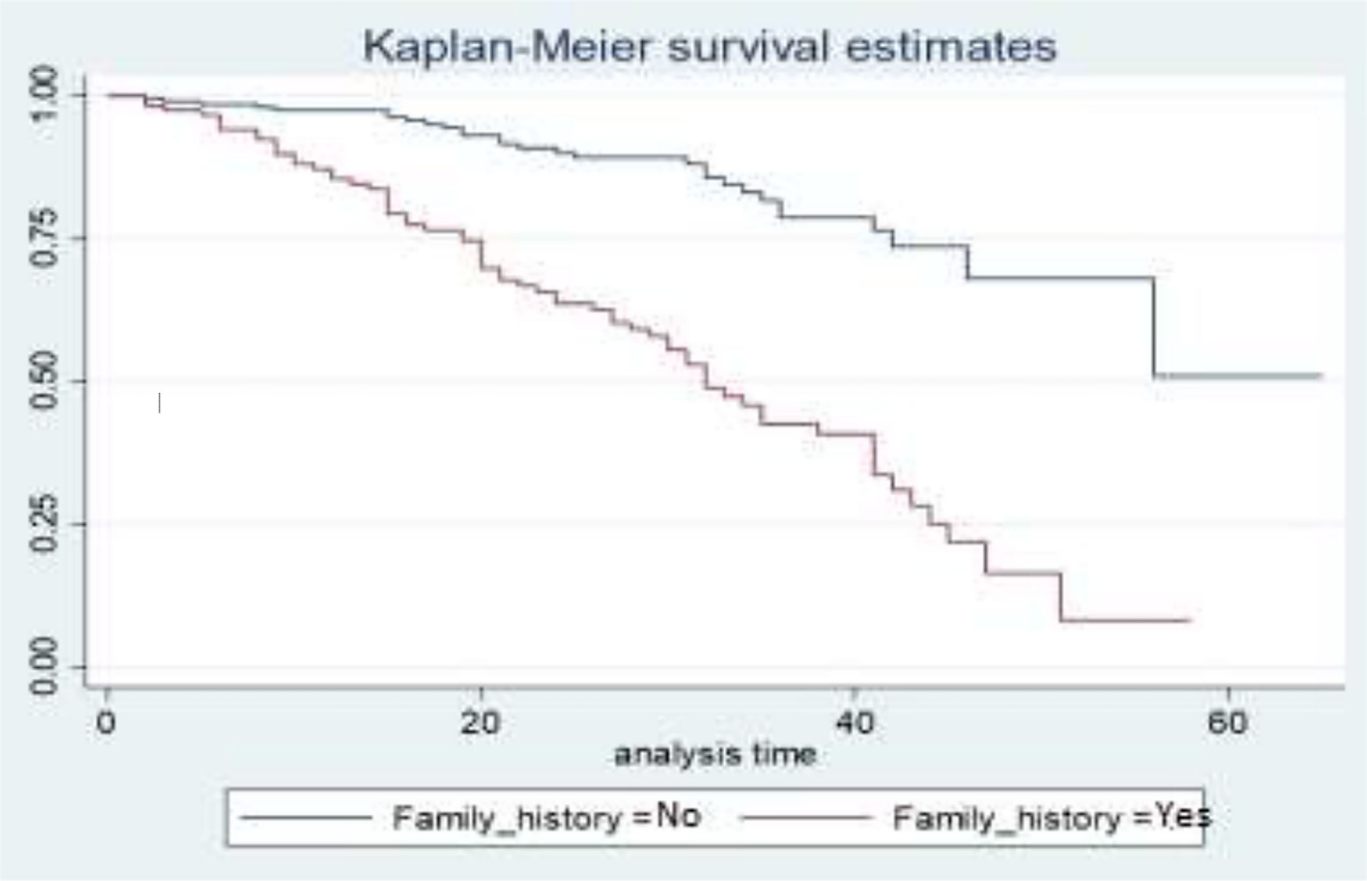
Survival curves for family history.

**Figure 5** depicts the family history status as a gap in their survival curves. BC patients who have a BC history in their family have a lower survival experience compared to those who have no BC history in their family.

### Log rank test

It is vital to do some statistical tests that will be used as an introduction to our subsequent findings. Here we start with the test of equality of probabilities across the different groups of categorical variables using the log-rank test to look at the significance of the difference among different factors. The null hypothesis to be tested is that there is no difference between the probabilities of an event occurring at any time point for each population. The results are summarized in **Table 4**.

**Table 4:** Log-rank test and mean survival time among the different groups of covariates.

Variable	Categories	Mean of survival time	Median of Survivaltime	CI (95%) for mean		Log-rank test	
				LB	UB	Chi-2	P>|z|
**Residence**	Rural	32.3	33.00	29.4	35.2	38.2	0.000
	Urban	51.4	–	47.2	55.6		
**Stage**	1	45.4	–	44.2	46.6	53.9	0.000
	2	50.7	–	46.7	54.7		
	3	35.0	36.00	31.2	38.8		
	4	33.6	34.00	29.0	38.1		
**Complication**	No	59.7	–	55.3	64.0	80.6	0.000
	Yes	32.0	32.00	29.0	34.9		
**Treatment**	Chemotherapy	49.8	51.00	44.1	55.5	26.1	0.000
	Chemo&Surgery	34.4	34.00	30.4	38.5		
	Chemo&Hormonal	42.7	42.00	37.9	47.6		
**Pathology**	Ductal carcinoma	48.4	–	45.2	51.6	39.6	0.000
	Lobular carcinoma	33.9	35.00	30.1	37.8		
**Metastasis**	No	54.5	–	50.1	58.9	34.7	0.000
	Yes	34.9	36.00	31.9	37.9		
**Blood type**	A	48.0	51.00	43.2	52.8	9.8	0.020
	B	37.5	43.00	32.9	41.9		
	O	43.8	56.00	36.8	50.9		
	AB	39.7	41.00	35.4	44.0		
**BMI**	Obese	48.7	–	36.8	60.6	1.2	0.745
	Overweight	39.9	43.00	34.9	44.9		
	Normal	40.6	45.00	37.3	43.8		
	Underweight	39.8	44.00	36.5	43.1		
**Family history**	No	52.7	–	48.3	57.0	50.6	0.000
	Yes	31.9	32.00	28.6	35.3		
**Recurrent**	No	50.8	56.00	46.4	55.1	24.5	0.000
	Yes	35.4	36.00	31.8	38.9		

**Table 4** showed the mean and median survival times of each covariate, and the different groups of residence, stage, complication, treatment, pathology, metastasis, blood type, family history, and recurrence had a statistically significant difference in survival probabilities. On the other hand, the survival functions of the BMI categories were statistically insignificant. That means there is no significant difference between obese, overweight, normal, and underweight. The overall mean and median estimated survival times of breast cancer patients under study were 43.7 and 45 months, respectively.

### Cox proportional hazards model

#### Univariable analysis

In any data analysis, it is always a great idea to do some univariable analysis before proceeding to more complicated models. Single-covariate Cox proportional hazards model analysis is an appropriate procedure that is used to screen out potentially important variables before directly including them in the multivariable model. In the univariable analysis, the relationship between each covariate and the survival time of BC patients was examined. This result showed that age, residence, stage, complication, treatment, pathology, metastasis, blood type, family history, and recurrence are statistically significant at a 25% significance level. But the covariate BMI is not statistically significant at the modest level of significance of 25%.

### Multivariable Cox proportional hazards model

For our data, the multivariable Cox proportional hazards model was fitted by including all the covariates significant in the univariable analysis at a 25% level of significance. Covariates that became insignificant in the multivariable analysis were then removed one by one from the model starting with the largest p-value by using the purposeful variable selection technique. Accordingly, age, stage, complications, treatment, metastasis, blood type, and recurrence were included.

The multivariable analysis of survival time of BC patients using the Cox proportional hazard model is presented in **Table 2**, and it indicates that the parameter estimates of coefficients for the covariates with the associated standard error, hazard ratio, 95% confidence interval for the hazard ratio, and the p-value. A hazard ratio of less than one indicates that the covariates decrease the risk of death in BC patients, and greater than one indicates that the covariates increase the risk of death in BC patients. Therefore, covariates like age, stage, complication, metastasis, blood type, and recurrence decreased the risk of death in BC patients, whereas covariate treatment increased the risk of death in BC patients.

### Multivariable analysis of the AFT model

Multivariable AFT models of the Weibull, log-logistic, and log-normal distributions were fitted by including all the covariates that were significant in the univariable analysis at a 25% level of significance. The common applicable criteria to select the model is the AIC and Bayesian information criterion (BIC). Based on AIC and BIC, a model having the minimum AIC and BIC values was preferred. Accordingly, the Weibull AFT model (AIC = 310.310, BIC = 370.703) was found to be the best for the set of the given alternatives (see **Table 5**).

**Table 5:** Comparison of AFT models using AIC and BIC for a given dataset.

Baseline AFT distributions	AIC	BIC
Weibull	310.310	370.703
log-logistic	319.295	379.688
lognormal	320.914	381.306

The results of the Weibull multivariate AFT model show that the predictor covariates age, stage, complication, metastasis, blood type, family history, and recurrence was significantly associated with the survival of BC patients at a 5% level of significance. The remaining variables that were used in the univariable covariate analysis, such as residence, treatment, and pathology were not significantly associated with the survival of BC patients at a 5% level of significance (see **Table 6**). This implies that the covariate residence, treatment, and pathology, were not jointly associated with the survival of BC patients but rather had individual effects.

**Table 6:** Summary result for Weibull accelerated failure time model.

Variable	Categories	Coef.	*ф*	St. Error	P>|z|	95%CI
**Age**		−0.013	0.987	0.007	0.047	(0.975, 0.999)
**Stage**	1 (ref) 2 3 4	−0.788 −1.458 −1.356	0.455 0.233 0.258	−0.558 −0.545 −0.547	0.158 0.007 0.013	(0.152, 1.357) (0.080, 0.677) (0.088, 0.752)
**Complication**	No (ref) Yes	−0.823	0.444	−0.216	0.000	(0.291, 0.677)
**Treatment**	Chemotherapy (ref) Chemo&Surgery Chemo&Hormonal	0.086 0.242	1.089 1.274	0.145 0.172	0.554 0.158	(0.820, 1.447) (0.910, 1.783)
**Pathology**	Ductal carcinoma (ref) Lobular carcinoma	−0.164	0.849	0.125	0.189	(0.664, 1.084)
**Metastasis**	No (ref) Yes	−0.291	0.747	0.147	0.047	(0.560, 0.997)
**Blood type**	A (ref) B AB O	−0.138 −0.435 −0.029	0.871 0.647 0.972	0.189 0.210 0.192	0.463 0.038 0.882	(0.602, 1.260) (0.429, 0.976) (0.668, 1.415)
**Family history**	No (ref) Yes	−0.312	0.732	0.140	0.026	(0.556, 0.963)
**Recurrent**	No (ref) Yes	−0.324	0.724	0.121	0.007	(0.571, 0.917)

AIC = 310.310, BIC = 370.703.

In the AFT model, the sign of the coefficient indicates how a covariate affects logged survival time. Thus, a positive coefficient increases the logged survival time and, hence, the expected duration. A negative coefficient decreases the logged survival time and, hence, the expected duration. Therefore, from **Table 6**, age, stage, complication, metastasis, blood type, family history, and recurrence had a negative coefficient and decreased the logged survival time as compared to their reference category.

The accelerated factor for the patient is 0.987 with 95% CI (0.975, 0.999) with a p-value of 0.047, indicating that age was a significant factor to determine the survival time of BC patients at a 5% level of significance. The accelerated factor for the patient is 0.233 and 0.283 with 95% CI (0.080, 0.677) and (0.088, 0.752) with P-values 0.007 and 0.013 for stages-III and IV, respectively, indicating that the patients whose stage was stages-III and IV had shortened the survival time from that of the patients whose stage is stage I. The accelerated factor for the patient is 0.444 with a 95% CI (0.291, 0.677) and a p-value of 0.000, indicating that the patients who had complicated diseases had a shorter survival time than those who had no complications.

The acceleration factor for the patient is 0.747 with a 95% CI (0.560, 0.997) with a p-value of 0.047 indicating that patients who had metastasized cancer shortened their survival time compared to those who had not metastasized cancer. The acceleration factor for the patient is 0.724 with 95% CI (0.571, 0.917) (P-value<0.05).

### Parametric shared frailty model results

Three AFT models were fitted and compared to analyze the survival times of BC patients and identify baseline distributions by using Akaike information criteria and Bayesian information criteria to identify associated risk factors. As a result, the Weibull accelerated failure time model, which has a smaller AIC (AIC = 310.310) and BIC (BIC = 370.703), was used as a baseline distribution for the parametric shared frailty model. To model the heterogeneity (random component) using a residence as a frailty term and investigate risk factors associated with the survival time of BC patient’s gamma shared frailty and inverse Gaussian shared frailty models with Weibull baseline distribution were used. The effect of the random component (frailty) was significant for both Weibull gamma shared frailty models and for Weibull inverse Gaussian shared frailty models. The AIC and BIC values for both parametric shared frailty models are summarized in **Table 7**. The Weibull gamma shared frailty model has a minimum AIC (309.932) value and a minimum BIC (377.875) value. This indicates the Weibull gamma shared frailty model was an appropriate model to describe BC patients’ dataset.

**Table 7:** Comparison of Weibull Gamma frailty and Weibull Inverse-Gaussian frailty model.

Distribution	AIC	BIC
Weibull Gamma frailty	309.932	377.875
Weibull Inverse-Gaussian frailty	310.021	380.635

The three models—Cox proportional hazards model, the accelerated failure time model, and the parametric shared frailty model—were included in this study to know the relationship between the survival time of BC patients and the covariates. The interpretation of the Cox proportional hazards model was based on hazard ratio; that of the accelerated failure time model was based on time ratio; and that of the frailty model had an additional unobserved heterogeneity or unmeasured risk factor. To construct the final model that best fits BC patients’ dataset, we made a comparison using AIC and BIC. From **Table 8**, we can see the values of AIC and BIC for the three models. A lower value of AIC and BIC suggests a better model fits the data. The Weibull gamma shared frailty model, which has a lower value than AIC (1,064.96) and BIC (1,064.96), appears to be an appropriate model compared with other models.

**Table 8:** Comparison of Cox PH, Weibull AFT, and Weibull gamma shared frailty model.

Models	AIC	BIC
Cox	799.327	852.171
Weibull AFT	310.310	370.703
Weibill gamma shared	309.932	377.875

### Weibull gamma shared frailty model result

This model is the same as the Weibull AFT model discussed in the previous section, except that a frailty component has been included. The frailty in this model is assumed to follow a gamma distribution with a mean of 1 and variance equal to theta (θ). The variability (unobserved heterogeneity) in the residence estimated by the selected model (Weibull-gamma shared frailty model) is θ = 0.002. A variance of zero (θ = 0) would indicate that the frailty component does not contribute to the model. A likelihood ratio test for the hypothesis θ = 0 is shown in **Table 2** below and indicates a chi-square value of 3.17 with one degree of freedom, resulting in a highly significant p-value of 0.000. This implied that the frailty component had a significant contribution to the model. The associated Kendall’s tau (τ), which measures dependence within clusters (residence), is estimated to be 0.0001. The estimated value of the shape parameter in the Weibull gamma shared frailty model is 1.941 (p = 1.941). The Weibull shape parameter greater than one indicates the hazard of failure increases with time.

All categorical variables were significant except for one category of pathology type. The confidence intervals of the acceleration factor for all significant categorical covariates do not include one at a 5% level of significance. This shows that they were significant factors in the survival of BC patients in Ethiopia. However, from the variable of pathology type category, pathology category lobular carcinoma using ductal carcinoma as the reference category with (p-value = 0.268, *ф* = 0.8971, 95%CI = 0.682, 1.112) were not significantly associated with the survival of BC patients. Accordingly, BC patients who came from urban areas (*ф* = 1.300) had a prolonged survival time when compared to BC patients who came from rural areas. From BC patients who get chemotherapy and surgery (*ф* = 1.125), BC patients who get chemotherapy and hormonal therapy (*ф* = 1.386) had prolonged survival time when compared to BC patients who get only chemotherapy.

The 95% confidence interval for the acceleration factor of age was 0.974, 0.999. This confidence interval does not include one, and the p-value was small (p-value = 0.032), indicating that age was a significant factor determining the survival time of BC patients at a 5% level of significance. Accordingly, the acceleration factor for age was less than one. This shows that a one-unit increase in age shortened the survival time of BC patients by a factor of 98.7% (**Table 9**). The acceleration factor and its 95% confidence interval for residence were 1.128 and (1.012, 1.672), respectively. This shows the survival time of BC patients who came from rural areas was shorter when compared to BC patients who came from urban areas (**Table 9**).

**Table 9:** Results of Weibull gamma shared frailty model.

Variable	Categories	Coef.	*ф*	S.E (cof.)	P>|z|	95% CI (*ф*)
**Age**		−0.014	0.987	0.006	0.032	(0.974, 0.999)
**Residence**	Rural (ref) Urban	0.263	1.128	0.128	0.040	(1.012, 1.672)
**Stage**	1 (ref) 2 3 4	−0.841 −1.495 −1.318	0.430 0.224 0.268	0.553 0.540 0.540	0.127 0.006 0.015	(0.146, 1.271) (0.078, 0.646) (0.093, 0.772)
**Complication**	No (ref) Yes	−0.789	0.454	0.212	0.000	(0.299, 0.689)
**Treatment**	Chemotherapy (ref) Chemotherapy and Surgery Chemotherapy and Hormonal	0.117 0.326	1.125 1.386	0.143 0.177	0.411 0.005	(0.850, 1.488) (0.079, 0.959)
**Pathology**	Ductal carcinoma (ref) Lobular carcinoma	−0.138	0.871	0.125	0.268	(0.682, 1.112)
**Metastasis**	No (ref) Yes	−0.138	0.726	0.146	0.029	(0.544, 0.967)
**Blood type**	A (ref) B AB O	−0.086 −0.450 0.043	0.918 0.638 1.044	0.188 0.205 0.192	0.649 0.028 0.821	(0.634, 1.329) (0.427, 0.953) (0.717, 1.522)
**Family history**	No (ref) Yes	−0.261	0.770	0.139	0.001	(0.586, 0.912)
**Recurrent**	No (ref) Yes	−0.346	0.707	0.119	0.004	(0.559, 0.894)

P = 1.941 σ = 0.515 θ = 0.0002, AIC = 309.932 BIC = 377.885.

LR test of theta = 0 chibar2 (01) = 1.0 pro ≥chibar2 = 0.00.

θ, variance of the random effect; p, shape parameter; σ, scale parameter; AIC, Akaike information criteria; LR, likelihood ratio; prob, probability; chibar2, Chi-square; chibar2(01), Chi-square distribution with 0 and 1 degrees of freedom.

The acceleration factor and its 95% confidence interval for stage-III BC patients were estimated to be 0.224 and (0.078, 0.646), respectively, and for stage-IV BC patients, they were estimated to be 0.268 and (0.093, 0.772), respectively. However, for both categories of stages III and IV of BC patients, the acceleration factor was less than one, indicating that both categories of stages III and IV shortened the survival time of BC patients as compared to stage-I (**Table 9**).

The 95% confidence interval for the acceleration factor of a covariate complication was (0.260, 0.616) for those in the category of having complications compared to those with no complications. Accordingly, the acceleration factor for complications was less than one. This study showed that having a complication shortened the survival time of BC patients by a factor of 45% when compared with having no complication (**Table 9**).

The acceleration factor and its 95% confidence interval for treatment were estimated to be 0.326 and (0.079, 0.959), respectively. The confidence interval did not include one, and the p-value was 0.05, indicating that treatment was a significant factor in determining the survival time of BC patients at a 5% level of significance. Accordingly, for the categories of chemotherapy and hormonal therapy, the acceleration factor was greater than one, indicating that the categories of gating both chemotherapy and hormonal therapy prolonged the survival time of BC patients as compared to gating only chemotherapy treatment (**Table 9**).

The 95% confidence interval for the acceleration factor of metastasis status of cancer was (0.544, 0.967) for the category metastasize cancer (not metastasize cancer as a reference category). This confidence interval does not include one, and the p-value was small (p-value = 0.029), indicating that metastasis was a significant factor in determining the survival time of BC patients at a 5% level of significance. However, the acceleration factor for metastasis was less than one. This shows that having a metastasized cancer type shortened the survival time of BC patients by a factor of 72% when compared with not having a metastasized cancer type (**Table 9**).

The acceleration factor and its 95% confidence interval for blood type were estimated to be 0.638 and (0.427, 0.953), respectively. The confidence interval did not include one, and the p-value was small (p-value = 0.029), indicating that blood type was a significant factor in determining the survival time of BC patients at a 5% level of significance. Accordingly, for the categories of having blood type AB, the acceleration factor was less than one, indicating that the categories of having blood type AB shortened the survival time of BC patients as compared to those having blood type A (**Table 9**).

The 95% confidence interval for the acceleration factor of family history status was (0.586, 0.912) for the category having a family history of BC. This confidence interval does not include one, and the p-value was very small (p-value = 0.001), indicating that family history was a significant factor in determining the survival time of BC patients at a 5% level of significance. Accordingly, the acceleration factor for family history was less than one (0.770). This shows that having a family history of cancer shortened the survival time of BC patients by a factor of 77% when compared with BC patients who did not have a BC history in their family (**Table 9**).

The acceleration factor and its 95% confidence interval for recurrence was estimated to be 0.707 and (0.559, 0.894), respectively. The confidence interval did not include one, and the p-value was very small (p-value = 0.004), indicating that recurrence was a significant factor in determining the survival time of BC patients at a 5% level of significance. However, for the recurrent cancer type, the acceleration factor was less than one, indicating that the recurrent cancer type has shortened the survival time of BC patients as compared to those not having the recurrent cancer type (**Table 9**).

## Discussion

The main goal of the study was to identify predictors of BC patients who were treated in Bahir Dar comprehensive specialized hospital using survival analysis models. The covariates that were included in the study were age, residence, stage, complications, treatment, pathology, metastasis, blood type, BMI, family history, and recurrence. The outcome variable of interest was the survival time of BC patients measured in months.

In all multivariable analyses of the Cox proportional hazards model and the AFT model, all relevant factors from the univariable analysis were taken into consideration. AIC was used for model comparison, and the model with the lowest AIC values was chosen as the best (45). For BC patient datasets, the Weibull gamma shared frailty model performed the best among AFT models. The factors of age, residence, stage, complication, treatment, metastasis, blood type, family history, and recurrence were substantially linked with the survival time of BC patients in the Weibull gamma-shared frailty model. Due to the flexibility of its hazard function and mathematical tractability, the gamma distribution is chosen for the frailty term (46). The residence effect was significant (p-value = 0.000) in the Weibull-Gamma shared frailty model. This showed that there was heterogeneity among residents regarding the survival times of BC patients.

The result of the study showed that age was associated with the survival time of BC patients at a 5% level of significance. The risk of death increases with the increasing age of BC patients. This result was in line with the study done by (47) that found that age is highly associated with the survival time of BC patients. This result was also consistent with the study conducted by (20); the result showed that increasing the age of BC patients significantly increased the risk of death due to BC. Also, the study done by (31) at the Ayder comprehensive specialized hospital in Tigray, Ethiopia, is in line with this study.

In this study, the variable residence significantly affects the survival time of BC patients. The results show that BC patients who come from urban areas have higher survival rates as compared to patients who come from rural areas. For the urban category, the acceleration factor is greater than one. The categories of urban prolonged the survival time of BC patients as compared to its reference. This result is similar to the (21) report that BC patients whose residence in a rural area increases the mortality of BC patients. This result was also consistent with the study conducted by (31) at the Ayder comprehensive specialized hospital in Tigray, Ethiopia.

The findings of this study, the stage of BC patients significantly affected their survival of BC patients. The result shows that the category of stages III and IV harmed the survival of BC patients. Since, for both categories of the stage, the acceleration factor is less than one, being on stages III and IV shortens the survival of BC patients as compared to stage-I. This means that BC patients who came for treatment at an early stage have a better survival rate than patients who came at an advanced stage. Similar findings by (46) indicate that advanced-stage BC patients are more likely to experience the event (death). This result was also consistent with the study conducted by (48) in western Amhara, Ethiopia.

The result of the study suggested that the variable complication significantly affects the survival time of BC patients. Having complicated diseases like anemia, diabetes, and hypertension shortens the survival time of BC patients. That means patients who have no associated diseases like anemia, diabetes, or hypertension are more likely to have a higher survival rate as compared to BC patients who have complications. This finding supports the result of (45). The study conducted by (49) is in line with this study.

This study shows that treatment has a statistically significant effect on the survival time of BC patients. Since, for the category of chemotherapy and hormonal therapy, the accelerated factor is greater than one, rather than gating only chemotherapy treatment, gating both chemotherapy and hormonal therapy treatment increases the survival time of BC patients. In line with (50), for breast cancer patients, in addition to chemotherapy, using additional treatments like surgery, radiotherapy, or hormonal therapy is likely to reduce the patient’s death.

In this study, a prognostic factor such as the stage of breast cancer had a significant effect on patient status. When cancer has metastasized, the patient’s chance of survival is reduced by 72.6% compared to when the cancer has not spread. According to a related American study (19), metastatic cancer tends to shorten the survival time or raise the fatality rate of BC patients. Compared to their reference blood type, BC patients with blood type AB had a shorter survival period. According to (51), the blood group should also be considered in addition to these risk factors when determining a patient’s prognosis, according to (51).

The findings of this study also found that family history determines the survival of BC patients. Patients who have a breast cancer history in their family are more likely to die earlier as compared to patients who have no breast cancer history in their family. This is consistent with the results of several other studies (18) that found that there is no evidence for a relationship between a family history of breast cancer and survival time.

The results of this study also suggested that variable recurrence was a significant predictive factor for the survival time of BC patients. That means BC patients who have recurrent cancer have a shorter survival time than patients who do not have recurrent cancer types.

### Limitations of the study

This study is based on cross-sectional data and hence does not assess the prevalence of breast cancer over time. The retrospective analysis causes inevitable bias, and no external data sets were used for validation. Besides, other socioeconomic, demographic, biological, and behavioral characteristics were not considered. Thus, we authors would like to recommend that future researchers consider these characteristics as they might affect breast cancer patients.

## Conclusions

The overall mean and median estimated survival times of breast cancer patients under study were 43.7 and 45 months, respectively. The findings of this study revealed that the residence effect between rural and urban areas was significant in describing the survival times of breast cancer patients. Therefore, the effects of this unobserved heterogeneity were included in the model. The Weibull-Gamma shared frailty model is the most appropriate model among the Weibull-Inverse-Gaussian shared frailty models for the survival time of breast cancer patients. The study found results based on that multivariable. Patient characteristics such as age, stage, complication, treatment, metastasis, blood type, family history, and recurrence were the prognostic factors that determined the survival time of breast cancer.

## Data availability statement

The raw data supporting the conclusions of this article will be made available by the authors, without undue reservation.

## Ethics statement

Ethical review and approval were not required for the study on human participants in accordance with the local legislation and institutional requirements. Written informed consent for participation was not required for this study in accordance with the national legislation and the institutional requirements.

## Author contributions

BF proposed the first draft, conducted data analysis, and interpretation, and wrote the manuscript. LT and AM edit and revise the manuscript. All authors contributed to the article and approved the submitted version.
